# LapF and Its Regulation by Fis Affect the Cell Surface Hydrophobicity of *Pseudomonas putida*

**DOI:** 10.1371/journal.pone.0166078

**Published:** 2016-11-03

**Authors:** Andrio Lahesaare, Hanna Ainelo, Annika Teppo, Maia Kivisaar, Hermann J. Heipieper, Riho Teras

**Affiliations:** 1 Institute of Molecular and Cell Biology, University of Tartu, Tartu, Estonia; 2 Helmholtz Centre for Environmental Research—UFZ, Department of Environmental Biotechnology, Leipzig, Germany; Beijing Institute of Microbiology and Epidemiology, CHINA

## Abstract

The ability of bacteria to regulate cell surface hydrophobicity is important for the adaptation to different environmental conditions. The hydrophobicity of cell surface can be determined by several factors, including outer membrane and surface proteins. In this study, we report that an adhesin LapF influences cell surface hydrophobicity of *Pseudomonas putida*. Cells lacking LapF are less hydrophobic than wild-type cells in stationary growth phase. Moreover, the overexpression of the global regulator Fis decreases surface hydrophobicity by repressing the expression of *lapF*. Flow cytometry analysis revealed that bacteria producing LapF are more viable when confronted with methanol (a hydrophilic compound) but are more susceptible to 1-octanol (a hydrophobic compound). Thus, these results revealed that LapF is the hydrophobicity factor for the cell surface of *P*. *putida*.

## Introduction

The ability to regulate cell surface hydrophobicity is important for bacterial adaptation to different environmental conditions [1,2]. Despite the fact that hydrophobicity of cell surface regulates physiology only passively, the influence to competitiveness of bacteria is remarkable. Cell hydrophobicity dictates the interaction to biotic and abiotic surfaces, the consumption of aromatic compounds, the protection against toxic compounds and even the effectiveness of bacterial motility in soil [3–7]. Additionally, the hydrophobic bacterial cell surface can provoke aggregation of cells in soil [8] promoting degradation of aromatic compounds such as phenol, pyridine and its derivatives via various metabolic pathways [9]. The hydrophobicity of surface may also have a protective role for bacteria. For example, an increase of cell surface hydrophobicity protects bacteria against several kinds of environmental stressors including osmotic stress, heat shock, and solvents [2,7,10].

The hydrophobicity of bacterial cell surface can be determined by several factors such as outer membrane proteins, lipopolysaccharides, S-layer proteins, lipoteichoic acids, and fimbrial adhesins [11–14]. For instance, enteric bacteria use fimbriae for adhesion to host cells via interaction of fimbrial lectin to carbohydrate of host cells [15]. These fimbriae contain a high number of hydrophobic amino acids that probably help overcome the initial electrostatic repulsion barrier that exist between the host cell and the surface of bacteria [16,17].

*Pseudomonas putida* promotes plant growth by colonizing plant roots and protecting crop plants against pathogenic organisms [18]. Therefore, the adhesion and biofilm formation of *P*. *putida* has been investigated already for decades [19]. Colonization of plant roots starts with adhesion of bacteria to root surfaces. Once the initial weak interaction turns into irreversible attachment, bacteria start dividing and producing a biofilm matrix [18,20]. The biofilm matrix of *Pseudomonas putida* consists mainly of proteins [21,22]. Two large proteins LapA and LapF, which act as adhesins, were shown to be essential for *P*. *putida*`s biofilm formation [22–24]. It has been demonstrated that LapA is required for cell-surface interactions and is responsible for biofilm initiation [22,25], whereas LapF is participating in mature biofilm, providing cell-cell interactions [25,26]. In 2014, Boyd *et al*. showed that LapA can bind to hydrophilic as well as hydrophobic surfaces [27], but there has been no report so far linking LapF and hydrophobicity in *P*. *putida*.

The phenotypes needed for successful root colonization like motility, chemotaxis, endurance of oxidative stress, composition of outer membrane, quorum sensing and biofilm formation are complexly controlled by global regulators [18,28–30]. Therefore, it was no surprise that the global regulator Fis is involved in regulation of the migration, biofilm formation and competitiveness of *P*. *putida* on the barley roots [22,31,32]. Fis is a small DNA-binding homodimeric protein, which is participating in several important processes including regulation of transcription and recombination [33–36]. Fis is well-studied in enterobacteria where *fis* knock-out mutants are viable; however, in pseudomonads it seems to be an essential protein, as *fis* deletion is lethal [33,37–39]. Thus, for studying the involvement of Fis in regulation processes, the options are limited to using *fis* overexpression.

We have previously shown that *fis* overexpression enhances *P*. *putida* biofilm formation most probably caused by an increase in the abundance of LapA of about 1.6 times compared to *P*. *putida* wild-type cells [22,31]. However, we have seen that *fis* overexpression represses the amount of LapF about 4 times. The Fis binding site Fis-F2 is mapped 150 bp upstream of the *lapF* gene coding sequence and the binding of Fis to this sequence represses the transcription of *lapF* [32]. Therefore, it was intriguing to study whether the two largest adhesins of *P*. *putida* LapA and LapF take part in regulation of cell surface hydrophobicity, as it was previously shown that cells growing in biofilm are usually more hydrophobic [7,40,41].

In this study, we measured the cell surface hydrophobicity, analysed as water contact angles (θ_w_), of *P*. *putida* cells, when lacking the adhesins LapA and/or LapF. Whereas the absence of LapA had no effect, the lack of LapF significantly reduced the surface hydrophobicity in stationary-phase cells. In addition, the involvement of Fis in the regulation of *P*. *putida*`s hydrophobicity was shown, as Fis repressed the level of LapF in stationary phase and thereby decreased the hydrophobicity of *P*. *putida*. The effect of LapF on *P*. *putida*`s adaptive properties towards hydrophobic and hydrophilic compounds was studied by flow cytometry analysis.

## Materials and Methods

### Bacterial strains, plasmids, oligonucleotides and media

Bacterial strains and plasmids used in this study are described in Table 1 and oligonucleotides are described in Table 2. Bacteria were grown in complete LB medium. Antibiotics were added at the following concentrations: 10 μg gentamicin ml^ˉ1^, 200 μg streptomycin ml^ˉ1^, 50 μg kanamycin ml^ˉ1^, 1500 μg benzylpenicillin ml ^ˉ1^. *E*. *coli* was incubated at 37°C and *P*. *putida* at 30°C. Bacteria were electrotransformed as described by Sharma & Schimke [42]. *E*. *coli* strain CC118 λ*pir* [43] was used as a host strain for DNA cloning procedures and a donor strain in conjugation experiments.

**Table 1:** Bacterial strains and plasmids used in this study.

Strain or plasmid	Genotype or description	Source/reference
***E*. *coli***		
CC118 λ*pir*	Δ(*ara-leu*) *araD* Δ*lacX74 galE galK phoA20 thi-1 rpsE rpoB argE* (Am) *recA1 λpir* phage lysogen	[43]
***P*. *putida***		
PSm	PaW85, isogenic to KT2440; chromosomal mini-Tn*7*-ΩSm1 (Sm^r^)	[31]
F15	PaW85, isogenic to KT2440; chromosomal mini-Tn*7*-ΩGm-term-*lacI*^q^-P_*tac*_-*fis*-T1T2 (Gm^r^)	[31]
PSmΔ*lapA*	PSm; ΔPP0168 (Sm^r^)	[22]
PSmΔ*lapF*	PSm; ΔPP0806 (Sm^r^)	[22]
PSmΔ*lapA*Δ*lapF*	PSm; ΔPP0168 ΔPP0806 (Sm^r^)	[22]
F15Δ*lapA*	F15; ΔPP0168 (Gm^r^)	[22]
F15Δ*lapF*	F15; ΔPP0806 (Gm^r^)	[22]
F15Δ*lapA*Δ*lapF*	F15; ΔPP0168 ΔPP0806 (Gm^r^)	[22]
PSmKm	PSm; Km gene is located 355 bp upstream of *lapF* coding sequence (Sm^r^, Km^r^)	This study
F15Km	F15; Km gene is located 355 bp upstream of *lapF* coding sequence (Gm^r^, Km^r^)	This study
F15KmFm	F15; Fis binding site Fis-F2 is mutated (Gm^r^, Km^r^)	This study
PSmlapF3	PSm; native DNA of 200 bp (including Fis-F2 binding site) upstream of *lapF* gene is replaced by 1.9 kb DNA of *lacI*^q^-P_*tac*_ (Km^r^, Sm^r^)	This study
F15lapF3	F15; native DNA of 200 bp (including Fis-F2 binding site) upstream of *lapF* gene is replaced by 1.9 kb DNA of *lacI*^q^-P_*tac*_ (Km^r^, Gm^r^)	This study
**Plasmids**		
pSEVA-lacItac-1	RK2 expression vector containing in Ecl136II site a 1981-bp-long blunted BamHI fragment of P_*tac*_-promoter and *lacI*^q^ repressor from pBRlacItac (Amp^r^)	This study
pBRlacItac	Expression vector containing P_*tac*_-promoter and *lacI*^q^ repressor in pBR322 (Amp^r^)	[45]
pBluescript KS	*E*. *coli* cloning vector (Amp^r^)	Stratagene
pBlc-Fp	Cloning vector pBluescript KS containing in SmaI site a 438-bp-long region of *lapF* upstream and downstream DNA (Amp^r^)	This study
pBlc-Fm	Cloning vector pBluescript KS containing in SmaI site a 438-bp-long region of *lapF* upstream and downstream DNA with mutated Fis-F2 binding site (Amp^r^)	This study
pBlc-Fy	Cloning vector pBluescript KS containing in SmaI site a 506-bp-long region of *lapF* upstream DNA (Amp^r^)	This study
pBLKT-Fis-mut	177-bp-long promoter region of the *lapF* gene with the mutated Fis-F2 site cloned into pBLKT BamHI site (Km^r^)	[32]
pGP704-L	*Pir*-dependent R6K replicon suicide vector (Amp^r^)	[46]
pGP-Fm	Suicide vector pGP704-L containing in SacI and SalI sites a 438-bp-long region of *lapF* upstream and downstream DNA with mutated Fis-F2 binding site (Amp^r^)	This study
pGP-Fp	Suicide vector pGP704-L containing in SacI and SalI sites a 438-bp-long region of *lapF* upstream and downstream DNA (Amp^r^)	This study
pGP-Fy	Suicide vector pGP704-L containing in SacI and SalI sites a 506-bp-long region of *lapF* upstream DNA (Amp^r^)	This study
pGP-FmFy	Suicide vector pGP704-L containing in Ecl136II and XbaI sites a 506-bp and 438-bp-long regions upstream and downstream of *lapF* gene (Amp^r^)	This study
pGP-FpFy	Suicide vector pGP704-L containing in Ecl136II and XbaI sites a 506-bp and 438-bp-long regions upstream and downstream of *lapF* gene (Amp^r^)	This study
pGP-FpFy-Km	Suicide vector pGP704-L containing in Ecl136II and XbaI sites a 506-bp and 438-bp-long regions upstream and downstream of *lapF* gene, with Km resistance gene between them in SacI site (Amp^r^, Km^r^)	This study
pGP-FmFy-Km	Suicide vector pGP704-L containing in Ecl136II and XbaI sites a 506-bp and 438-bp-long regions upstream and downstream of *lapF* gene, containing mutated Fis-F2 binding site and with Km resistance gene between them in SacI site (Amp^r^, Km^r^)	This study
pGP-FpFy-Km-lacItac-lapFSD	Suicide vector pGP704-L containing in SalI and BglII sites a 506 bp and 527 bp long regions upstream and downstream of *lapF* gene, with Km resistance gene and *tac* promoter with *lacI* repressor gene. Shine-Dalgarno region replaced; Fis binding site Fis-F2 and the native promoter of *lapF* gene are deleted (Amp^r^, Km^r^)	This study
pUTmini-Tn*5* Km2	Suicide vector, source of Km resistance gene (Amp^r^, Km^r^)	[47]

**Table 2:** Oligonucleotides used in this study.

Oligonucleotidedesignation^*a*^	Oligonucleotide sequence^*b*^	Complementary region
fis-BamHI (BamHI)	5’-AGAGGATCCTTACAACAAGTCGTACTGC-3’	positions 321 to 303 in relation to the initiator codon of the *fis* gene (PP4821)
Km0	5’-GTGCAATGTAACATCAGAGATTTT -3’	positions -68 to -92 in relation to the initiator codon of the Km^r^ gene
LapF-fw (BglII)	5’-TAGATCTTTCGCTGAGGCTTTTCTAC-3’	positions 198 to 180 in relation to the initiator codon of the *lapF* gene (PP0806)
LapF-RACE1	5’-GCCGACGAAGACCATATC-3’	positions 241 to 224 in relation to the initiator codon of the *lapF* gene (PP0806)
LapF-rev (BglII)	5’-TTAGATCTCGGCGAAGAAGTTACCGATG-3’	positions 201 to 181 in relation to the initiator codon of the *lapF* gene (PP0806)
LapF-rev3 (BamHI)	5’-AGGATCCACCAGTTCGTCCAGCGAGG-3’	positions 311 to 329 in relation to the initiator codon of the *lapF* gene (PP0806)
LapF-rev4 (BamHI)	5’-AAGGATCCGGCGAAACCAGCAGGT-3’	positions 487 to 507 in relation to the initiator codon of the *lapF* gene (PP0806)
LapF-SacI (SacI)	5’-AAGAGCTCAGCGAAGCCCTAGCC-3’	positions -190 to -208 in relation to the initiator codon of the *lapF* gene (PP0806)
LapF-SD-SalI (SalI)	5’-AAAGTCGACAGAAGGTGGTTGTATGGACAACATCGTCGTCGC-3’	positions 0 to 20 in relation to the initiator codon of the *lapF* gene (PP0806)
LapI-fw	5’-CCTGGCTTGAGGTGATGTT-3’	positions 83 to 101 in relation to the initiator codon of the *lapI* gene (PP0804)
LapI-rev	5’-TGTGGCGGAGGATTTCATT-3’	positions 181 to 199 in relation to the initiator codon of the *lapI* gene (PP0804)
PP0806-2-rev (EcoRI)	5’-AAGAATTCGGCCTGTACCACGTCGG-3’	positions 728 to 710 downstream of the stop-codon of the *lapF* gene (PP0806)
PP0806-I-rev (EcoRI)	5’-AAGAATTCACCGCCTCAGCGTTTACC-3’	positions -685 to -676 in relation to the initiator codon of the *lapF* gene (PP0806)
Prtac	5’-AATTAATCATCGGCTCGTATAA-3’	positions -100 to -79 in relation to the initiator codon of the *fis* gene in the mini-Tn*7*-term-*lacI*^q^-P_*tac*_*-fis*-T1T2-ΩGm cassette
RpoDq-fw	5’-GCAACAGCAGTCTCGTATCA-3’	positions 15 to 34 in relation to the initiator codon of the *rpoD* gene (PP0387)
RpoDq-rev	5’-ATGATGTCTTCCACCTGTTCC-3’	positions 140 to 120 in relation to the initiator codon of the *rpoD* gene (PP0387)

^*a*^ restrictases are shown in brackets

^*b*^ restriction sites are underlined

To examine the growth parameters, the strains of *P*. *putida* were grown overnight in LB medium. These cultures were used to inoculate fresh LB media so that the absorbance of the cultures at 580 nm was approximately 0.1. The bacteria were grown in 96-well microtiter plates (150 μl media per well) and A_580_ was measured at 7 minute intervals using a Sunrise-Basic Tecan microplate reader (Tecan Austria GmbH, Austria). Approximately 150 viable count data points were produced for each growth curve. Growth rate (*μ*; h^−1^) and the length of lag-phase of bacteria were estimated from growth curves by the Gompertz model [44]. The mean of the growth rate of different strains was calculated from 6 parallels.

### DNA manipulations and strain construction

The plasmids used for the construction of strains PSmKm, F15Km and F15KmFm were obtained by several sequential cloning steps. At first, the 506-bp-long DNA region of *P*. *putida* chromosome locating 695 bp to 189 bp upstream of the *lapF* start-codon was amplified by using the primers PP0806-I-rev and lapF-SacI. Thereafter the PCR product was cloned into pBluescript KS vector opened by SmaI restrictase resulting in pBlc-Fy (Table 1). Secondly, the 438-bp-long DNA region of *P*. *putida* chromosome at positions 198 bp upstream to 240 bp downstream of the *lapF* start-codon, which contained Fis-F2 binding site was amplified by the primers lapF-fw and lapF-RACE1. The PCR product was cloned into the pBluescript KS vector opened by SmaI restrictase resulting in pBlc-Fp (Table 1).

For the construction of the strain F15KmFm two sequential PCRs were carried out to amplify the 438-bp-long DNA fragment containing mutated Fis-F2 Fis binding site. In the first PCR, the primers lapF-fw and lapF-down2 and the template plasmid pBLKT-Fis-mut [32] carrying mutated Fis-F2 site were used for the DNA amplification. In the second PCR, lapF-RACE1 and the product of the first PCR were used as primers for the DNA amplification of the *lapF* promoter region from the *P*. *putida* PSm chromosome. The obtained DNA fragment was inserted into the pBluescript KS vector opened by SmaI restrictase resulting in pBlc-Fm (Table 1).

**Fig 1:**
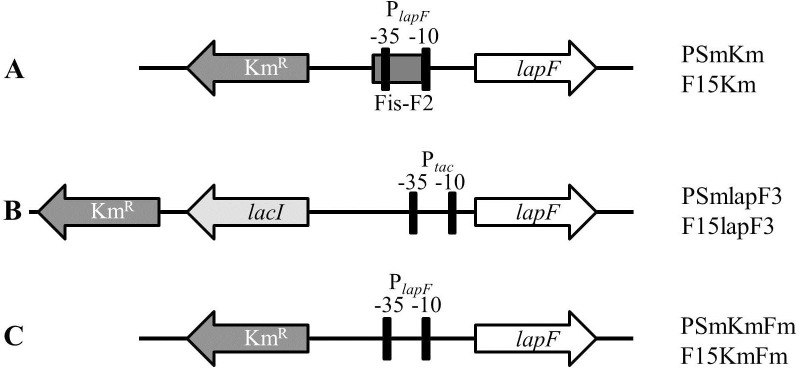
A scheme of the upstream regions of the *lapF* gene of *P*. *putida* strains constructed in this study. Striped box indicates Fis binding site, -35 and -10 promoter elements are marked with black boxes, genes and their directions are shown with arrows: *lapF* in white, *lacI* repressor gene in light grey and Km resistance gene in dark grey. P_*tac*_ indicates the IPTG-inducible promoter under the control of LacI repressor, and P_*lapF*_ marks the native promoter of the *lapF* gene. Strain names corresponding with the indicated genotypes are shown on the right. PSm refers to the wild-type and F15 to the *fis*-overexpression strains.

After that the plasmid DNA of pBlc-Fp and pBlcFm was cut with restrictases SacI and XhoI and 476-bp long DNA fragments, were cloned into pGP704-L opened by SacI and SalI restrictases, resulting in plasmids pGP-Fp and pGP-Fm, respectively (Table 1). Then the plasmid pBlc-Fy was cut with XbaI and EcoRV and the 552-bp-long DNA fragment was cloned into pGP-Fp and pGP-Fm opened with Ecl136II and XbaI restrictases, resulting in pGP-FpFy and pGP-FmFy, respectively (Table 1). Lastly, the Km-resistance gene from pUTmini-Tn*5* Km2 was cloned into pGP-FpFy and pGP-FmFy into SacI site resulting in pGP-FpFy-Km with native Fis binding site and pGP-FmFy-Km with mutated Fis binding site, respectively (Table 1). The constructed plasmids were introduced by electroporation into *E*. *coli* strain CC118 λ*pir*. The obtained donor-strains were mated with the helper plasmid-carrying strain *E*. *coli* HB101 and the *P*. *putida* recipient strains PSm or F15. Triple mating resulted in the strains PSmKm, F15Km (Fig 1A), PSmKmFm and F15KmFm (Fig 1C).

The plasmids used for the construction of the strains PSmlapF3 and F15lapF3 were obtained by two sequential clonings. At first, the P_*tac*_-promoter with the *lacI* repressor gene was cloned from pSEVA-lacItac-1 into pGP-FpFy-Km opened by XbaI restrictase resulting in pGP-FpFy-Km-lacItac (Table 1). Thereafter, the 438-bp-long DNA region containing the Fis-F2 binding site was replaced by the 527-bp-long DNA region (locates at the positions 20 bp upstream to 507 bp downstream of the *lapF* start-codon) amplified from the *P*. *putida* chromosome by using the primers lapF-SD-SalI and lapF-rev4 and cloned into the plasmid pGP-FpFy-Km-lacItac opened with restrictases SalI and BglII, resulting in the construct pGP-FpFy-Km-lacItac-lapFSD (Table 1). This plasmid carried downstream of the P_*tac*_ promoter a LacI-operator, a new artificial Shine-Dalgarno region and the first 240 nucleotides of *lapF* gene. The plasmid pGP-FpFy-Km-lacItac-lapFSD was introduced by electroporation into *E*. *coli* strain CC118λ*pir* and the triple mating was conducted as described previously. The obtained *P*. *putida* strains PSmlapF3 and F15lapF3 lacked the DNA region 200 bp upstream of the *lapF* start-codon including the *lapF* promoter elements and Fis-F2 binding site (Fig 1B). In these strains the *lapF* gene was transcribed under the control of IPTG-inducible P_*tac*_ promoter and the effects of Fis on the transcription of this gene were excluded.

All constructs were verified by the DNA sequencing in order to exclude PCR-generated errors in the cloned DNA-fragments. The accuracy of recombination in the desired *P*. *putida* strains was verified by the DNA sequencing of the relevant chromosomal regions of *P*. *putida*.

### Measurement of cell surface hydrophobicity

Cell surface hydrophobicity was analysed by the measurements of the contact angle, θ_w_, between water droplets and a filter (NC45, 0.45 μm pore size from Whatman, Maidstone, UK) covered with bacterial cells and mounted on a glass slide [10]. DSA100 drop shape analyser system from Krüss GmbH (Hamburg, Germany) was used for measurements of contact angles.

### Detection of the expression of *fis* and *lapF* in the constructed strains of *P*. *putida*

For the preparation of cell lysates, all studied *P*. *putida* strains were grown in 50 ml LB broth with or without 1 mM IPTG for 3 or 18 h. The cells were collected by centrifugation and lysed in RIPA buffer (50 mM Tris/HCl pH 7.4 buffer, 0.1% SDS, 1% NP-40, 1% Triton X-100, 0.5% DOC (sodium eoxycholate), 500 mM NaCl, 5 mM EDTA) and 100-fold diluted Halt protease and phosphatase single-use inhibitor cocktail (Thermo Scientific) as previously described [22]. The total amount of protein in the cleared supernatant was measured by the content of tryptophan and the proteins were separated in gradient (4–8%) polyacrylamide gel electrophoresis as previously described [22].

For the production of polyclonal mouse anti-Fis antibody, *P*. *putida* Fis(His) was overexpressed and purified with Ni-NTA agarose matrix (Qiagen) as previously described [37]. The polyclonal antibodies against Fis (PP4821) were produced and purified by LabAs. Western immunoblot analysis was carried out to detect the amount of Fis from the crude lysates of *P*. *putida*. Bacteria were grown to stationary-phase in LB medium in the presence or absence of 1 mM IPTG. The cells were collected by centrifugation and sonicated in Fis purification buffer (100 mM Tris/HCl, pH 7.5, 0.3 M NaCl, 5% v/v glycerol). The cell lysates were centrifuged at 12 000 g for 30 min at 4°C. The total amount of protein in the cleared supernatant was measured spectrophotometrically by the content of tryptophan [48]. Proteins were separated by Tricine-SDS-PAGE (10%) electrophoresis [49], transferred to a membrane and the membrane was probed with mouse anti-Fis as previously described [22].

### Flow cytometry analysis

*P*. *putida* grown for 18 h in LB medium was treated with different concentration of methanol and 1-octanol for 30 minutes at 30°C. The bacteriocidic level of methanol and 1-octanol are shown to be >50% and >0.05% respectively [50]. Therefore, methanol was added at final concentrations of 25 and 50% (v/v) and 1-octanol at 0.015 and 0.045% (v/v). Identical amounts of water were added to the control cells. Thereafter, the cells were stained using the LIVE/DEAD BacLight kit (Invitrogen). Staining of cells was performed as suggested by manufacturers and approximately 10,000 events from every sample were analysed with flow cytometer FACSAria (BD Biosciences).

### Quantitative real-time PCR

RNA for the qRT-PCR reactions was extracted from *P*. *putida* strains PSm and PSm*ΔlapF* with NucleoSpin^®^ RNA II kit (Macherey-Nagel) according to the manufacturer’s protocol. The SYBR Green qPCR assay was performed on the Rotor-Gene Q system (QIAGEN) using SuperScript^®^ III One-Step RT-PCR System with Platinum^®^ Taq (Invitrogen). The reaction mixture, which was optimized as previously described [51], contained 5 μl of SYBR Green Reaction Mix, 4 pmol of each primer, 10 ng of RNA, 0.2 μl of SuperScript^®^ III/ Platinum^®^ Taq Mix and RNase-free water for a total volume of 10 μl. The amplification program included the reverse transcription step at 50°C for 3 min and the initial denaturation at 96°C for 5 min followed by 40 cycles of 15 s at 95°C, 30 s at 62°C and 20 s at 72°C. SYBR Green fluorescence was measured after each extension step and the specificity of amplification was evaluated by the melting curve analysis. The *lapI* gene was amplified with the primers LapI-fw and LapI-rev and the reference gene *rpoD* with the primers RpoDq-fw and RpoDq-rev. RT-qPCR data was analysed with Rotor-Gene Q software version 2.0.2 and LinRegPCR program version 11 [52]. The amount of *lapI* mRNA in RT-qPCR was normalized against *rpoD* mRNA. The mRNA of *rpoD* was used as standard to reduce the random fluctuation of mRNA concentration. The fold difference between the *lapI* mRNA level and the *rpoD* mRNA level was estimated using the following formula:

$$\left(\frac{N_{0,lapI}}{N_{0,rpoD}}\right)=\left(\frac{E_{rpoD}^{Ct,rpoD}}{E_{lapI}^{Ct,lapI}}\right),\;\text{where}$$

N_0_ is the starting concentration of the amplicon, E the amplification efficiency and C_t_ the number of cycles needed to reach the threshold [52].

### Statistical analysis

The ANOVA and *post hoc* Bonferroni test at the significance level 0.05 were used to assess the variability of experimental data. The calculations were performed using Statistica 13 software.

## Results and Discussion

### LapF increased the hydrophobicity of *P*. *putida* cells

The hydrophobicity of cell surfaces of Gram-negative bacteria can be determined by several factors like outer membrane proteins, lipopolysaccharide composition and adhesins etc. [11–14]. It has been previously reported for *P*. *putida* that LapA is the key factor for the early attachment of cells to abiotic and biotic surfaces, and LapF is important for cell-cell interaction in mature biofilms [25,26]. Although LapA is required for the adhesion of bacterial cells to both the hydrophobic and hydrophilic surfaces [27], participation of this adhesin in affecting the hydrophobicity of the cell surface of *P*. *putida* has not been addressed prior to this work.

**Fig 2:**
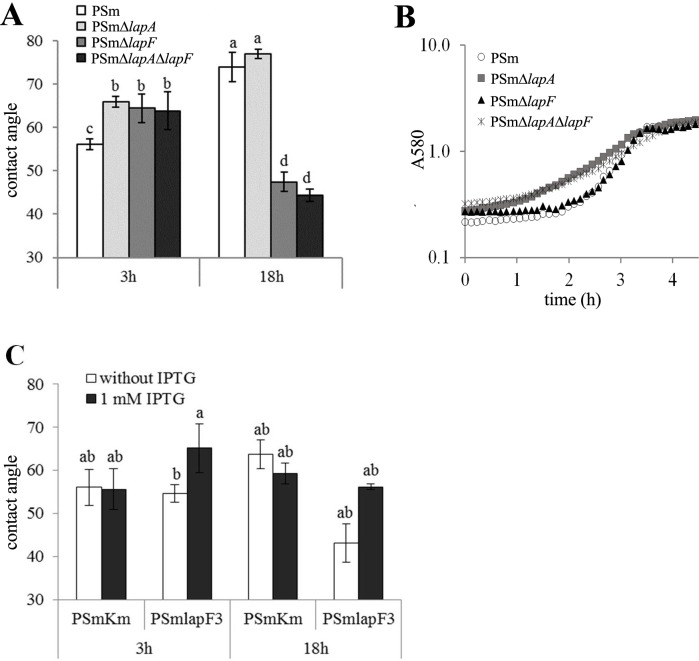
The cell surface hydrophobicity of *P*. *putida* PSm-originated strains. (A) Hydrophobicity of exponential phase (3h) and stationary-phase (18h) *P*. *putida* wild-type strain (PSm), the *lapA* knock-out mutant PSmΔ*lapA*, the *lapF* knock-out mutant PSm Δ*lapF* and the double knock-out mutant PSmΔ*lapA*Δ*lapF*. (B) The growth curve of PSm, PSmΔ*lapA*, PSmΔ*lapF* and PSmΔ*lapA*Δ*lapF* grown in LB medium. The growth curves of the first 4.5 hours are shown. Three independent biological experiments were done with similar results. Data from six parallels from one experiment is shown. (C) Hydrophobicity of exponential phase (3h) and stationary-phase (18h) *P*. *putida* strain expressing the *lapF* gene at natural level (PSmKm) and the strain PSmlapF3 expressing the *lapF* gene under the control of IPTG-inducible P_*tac*_ promoter grown in the presence or absence of 1 mM IPTG in LB medium. The level of cell surface hydrophobicity is given as water contact angles, θ_w_, (in degrees) between water droplets and a filter covered with bacterial cells. Error bars denote 95% confidence intervals of the means. Letters a–d depict statistical homogeneity groups according to ANOVA *post hoc* Bonferroni test. Data from at least eight independent measurements is shown.

In order to investigate whether *P*. *putida*`s two largest adhesins LapA and LapF could affect the hydrophobicity of cell surface, the cell surface hydrophobicity of PSmΔ*lapA*, PSmΔ*lapF* and the double-mutant PSmΔ*lapA*Δ*lapF* were compared with the wild-type strain PSm in both exponential and stationary growth phase. *P*. *putida* PSm wild-type cells harvested after 18 hours, in the stationary-phase, showed higher cell surface hydrophobicity (θ_w_ 76°, Fig 2A), which correlates with the previous findings that stressed bacteria have more hydrophobic cell surfaces [7]. Whereas stationary phase cells of the strain lacking LapA showed similar contact angles with the wild-type, the θ_w_ of the mutants lacking LapF (PSmΔ*lapF*) and the double mutant of LapA and LapF (PSmΔ*lapA*Δ*lapF*) decreased to 47° and 44°, respectively (Fig 2A).

Contrary to these observations, when cells were harvested in the exponential growth phase, all tested PSm knock-out mutants showed similar water contact angles varying between θ_w_ 64° and 66° (*P* = 1; Fig 2A). The cells of these mutants were approximately 1.2 times (*P*˂0.001) more hydrophobic than those of the wild-type cells (θ_w_ 56°, Fig 2A). As growth of bacteria can influence the result of cell surface hydrophobicity in exponential growth phase, we assessed the growth of *P*. *putida* PSm and the knock-out strains in LB medium (Fig 2B, Table 3). The strains grew similarly in stationary phase (data not shown), but differently in exponential growth phase. It is possible to distinguish two groups of strains by growth rate and lag-phase length: (i) PSm and PSmΔ*lapF*, and (ii) PSmΔ*lapA* and PSmΔ*lapA*Δ*lapF* (Fig 2B, Table 3). The growth parameters of PSm and PSmΔ*lapF* were similar and differed from *lapA* null-mutant strains (Table 3). Whereas the *lapF* expression is strongly repressed in exponentially growing *P*. *putida* [22,53], the cell surface hydrophobicity of PSm and PSmΔ*lapF* should be similar in exponential growth phase. Moreover, the absence of LapA in the surface of stationary phase cells did not affect the hydrophobicity of *P*. *putida* (Fig 2A). Thus, it seems that the cell surface hydrophobicity of knock-out mutants does not depend on neither LapF nor LapA in exponential growth phase. However, the important factor for increasing *P*. *putida* cell surface hydrophobicity in the stationary phase is LapF and not LapA.

**Table 3:** The growth parameters of *P*. *putida* strains grown in LB medium.

Strain	Growth rate, h^-1^	Lag-phase length, h
PSm	0.513 (0.033)* a	1.416 (0.089) a
PSmΔ*lapA*	0.415 (0.013) b	0.502 (0.089) b
PSmΔ*lapF*	0.483 (0.022) a	1.261 (0.164) a
PSmΔ*lapA*Δ*lapF*	0.398 (0.014) b	0.582 (0.180) b

* In the brackets are shown 95% confidence intervals of the means of 6 parallels. All *R* values were > 0.990. Letters a and b depict statistical homogeneity groups according to ANOVA *post hoc* Bonferroni test.

The protein hydrophobicity values in *P*. *putida* according to Kyte-Doolittle scale are -0.109 for LapA and 0.164 for LapF [54]. This scale is a measure for the hydrophobicity of amino acids [55], indicating that LapF is more hydrophobic than LapA. However, the total hydrophobicity of large proteins like LapA and LapF does not describe the surface properties of a protein. For example, LapA can bind to hydrophilic as well as hydrophobic surfaces [27]. According to the same database, two bacterial Ig-like domains are predicted in LapF locating in position 717 to 748 and 1158 to 1177 residues of amino acids [54]. Although, Ig-like domains are frequently found in the cell surface proteins of enterobacteria needed for adhesion, it is speculated that Ig-like domains are responsible for stabilization of protein rather than for adhesion [56]. For example, the Ig-like domains are found in the cytoplasmic enzymes SodC and LacZ that are not involved in adhesion [57]. Thus, the Ig-like domains in LapF probably stabilize the protein on the surface of *P*. *putida*.

It has been previously shown, that in *P*. *putida lapF* is expressed only in stationary growth phase and no LapF is detectable in exponential phase cells [22,53]. In order to study the involvement of LapF in cell surface hydrophobicity, *P*. *putida* strain PSmlapF3 was constructed, in which *lapF* expression was under the control of IPTG-inducible P_*tac*_ promoter (Table 1, Fig 1B). In addition, *P*. *putida* strain PSmKm was constructed as a reference strain for PSmlapF3 by inserting a Km resistance gene 355 bp upstream of the *lapF* gene (Table 1). This strain had the native *lapF* promoter, although the chromosome of *P*. *putida* was interrupted by Km^r^-gene insertion similarly to PSmlapF3 (Fig 1A and 1B).

**Fig 3:**
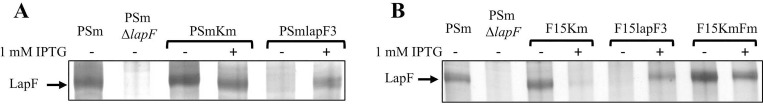
The presence of LapF in the cell lysates of *P*. *putida*. (A) Presence of LapF in the cell lysates of *P*. *putida* PSmKm and PSmlapF3; (B) Presence of LapF in the cell lysates of F15Km, F15lapF3 and F15KmFm. *P*. *putida* PSm and PSmΔ*lapF* were used as controls. Bacteria were grown for 18 hours in LB medium and total protein lysates were prepared. The supplementation of 1 mM IPTG is shown by”+” on the top of figure.

The expression of *lapF* in these newly constructed strains was confirmed with silver-stained SDS-polyacrylamide gels, where proteins from cell lysates were separated by gel-electrophoresis (Fig 3A). LapF was present in 18 h stationary PSmKm both in the absence and presence of IPTG (Fig 3A) similarly to strain PSm [22]. Thus, the insertion of the Km-resistance gene in front of the *lapF* gene did not affect the expression of *lapF*. On the contrary, in stationary-phase cells of PSmlapF3, the *lapF* expression was under the control of IPTG as LapF was detected only in the presence of IPTG (Fig 3A). The expression of *lapF* in exponentially growing bacteria was not detectable due to technical reasons. The lysates of 3 h grown exponential cells decomposed in SDS-gels probably due to high level of protease activity in cell lysates despite of the addition of protease inhibitor cocktail during cell lysate preparation (data not shown).

The cell surface hydrophobicities of PSmKm and PSmlapF3 were measured in exponential and stationary cells both in the presence or absence of 1 mM IPTG (Fig 2C). The stationary-phase PSmlapF3 cells cultivated in the presence of 1 mM IPTG showed 1.3 times (*P*˂0.001) higher contact angles (θ_w_ 56°) comparing to PSmlapF3 grown without IPTG (θ_w_ 43°, Fig 2C). In addition, the contact angles of PSmlapF3 with 1 mM IPTG (θ_w_ 59°) were comparable with that of PSmKm (*P* = 1). This proved that the transcription of the *lapF* gene from the P_*tac*_ promoter in the presence of IPTG can re-establish the hydrophobicity of *P*. *putida* cells to the level of the wild-type strain (Fig 2C). In exponential cells, when IPTG was added to the wild-type strain PSmKm, there was no significant change in the contact angles, as the θ_w_ value was approximately 56° for cells without IPTG as well (Fig 2C). However, the contact angle of *P*. *putida* cells increased 1.2 times (*P*˂0.001) already in the exponential phase when the transcription of the *lapF* gene was induced in PSmlapF3 by IPTG (θ_w_ 65°) if compared to the contact angle of PSmlapF3 grown without IPTG (θ_w_ 54°) or wild-type PSmKm (θ_w_ 56°, Fig 2C). The increase in hydrophobicity of PSmlapF3 exponentially growing cells in the presence of IPTG implied that LapF is responsible for the increase in cell surface hydrophobicity of *P*. *putida*. These results further confirmed the hypothesis that the expression of LapF increases the level of hydrophobicity of *P*. *putida* cells.

### Knocking-out the *lapF* gene did not influence the expression of the rest of genes in the *lapFHIJ* operon

**Fig 4:**
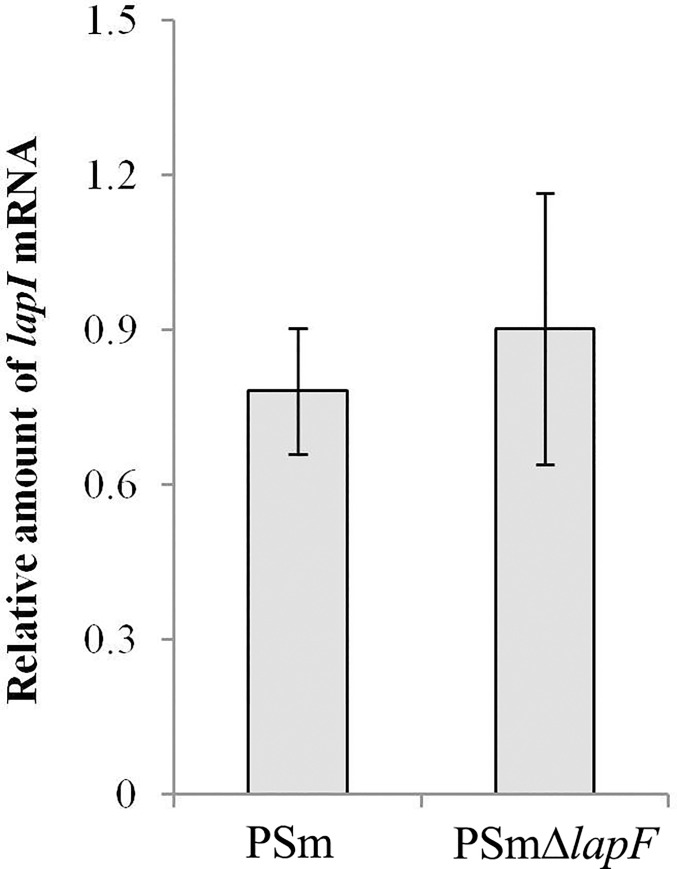
The relative amount of *lapI* mRNA. The amount of *lapI* mRNA relative to the *rpoD* reference mRNA in the wild-type and the Δ*lapF* strain, analysed by qRT-PCR.

To verify that the expression level of the other *lapFHIJ* operon genes has not changed by knocking out *lapF* gene, the *lapI* (PP_0804) mRNA level was measured with RT-qPCR (Fig 4). The mRNA level of *lapI* compared against the mRNA level of the reference gene *rpoD* was statistically insignificant (*P* = 0.3) between the wild-type and Δ*lapF* strain. Thus, the hydrophobicity of *P*. *putida* was most likely influenced by the *lapF* and not by the rest of the genes from the *lapFHIJ* operon.

### Fis overexpression reduced the hydrophobicity of stationary-phase cells by decreasing the level of LapF

The expression of *lapF* is controlled by two regulators. In stationary-phase cells, *lapF* expression is triggered by RpoS [26], the sigma factor required for starvation and general stress responses. The expression of *lapF* is additionally repressed by the global regulator Fis, which binds *lapF* promoter [32], resulting in the decline of the *lapF* expression [22].

To study the correlation between the cellular amount of Fis and hydrophobicity of *P*. *putida* cells, three strains originated from the *fis* overexpression strain F15 were constructed. First, the reference strain F15Km was constructed similarly to the PSmKm strain, which contained the native promoter in front of the *lapF* gene with the Fis binding site Fis-F2 (Table 1; Fig 1A). In addition, the artificial *lapF* expression strain F15lapF3 was constructed with the possibility to express the global regulator Fis and LapF by IPTG (Table 1; Fig 1B). Since the native promoter of the *lapF* gene was replaced by the *lacI-*P_*tac*_ cassette, we expected that the transcription of the *lapF* gene in this strain could be affected by the presence of IPTG but not by the level of *fis* overexpression. The third constructed *fis* overexpression strain F15KmFm carried a mutated Fis binding site Fis-F2 upstream of the *lapF* gene in *P*. *putida* chromosome (Table 1; Fig 1C). In this strain, the substituted nucleotides in the Fis binding sequence Fis-F2 reduced the Fis binding to the *lapF* promoter *in vitro* as described previously [32] and therefore *fis* overexpression should regulate *lapF* transcription modestly or not at all.

**Fig 5:**
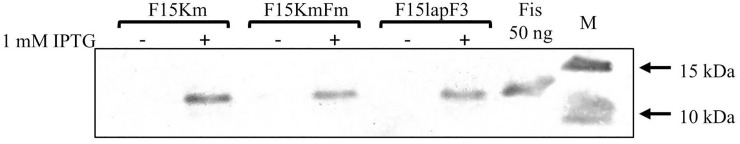
Overexpression of *fis* in *P*. *putida* cells determined by immunoblotting using polyclonal anti-Fis antibodies. Immunoblot analysis was performed with crude cell lysates prepared from *P*. *putida* strain F15 grown in LB medium for 18 h. The supplementation of 1 mM IPTG is shown by “+” above the lane. Thirty micrograms of crude cell lysate were analysed. Fifty nanograms of purified Fis (6His) was used as a positive control. Arrows show the location of marker proteins 15 and 10 kDa in size in the marker (M) lane.

The expression of *fis* and *lapF* in these F15-originated strains was assessed. The overexpression of *fis* was confirmed by the immunoblotting of cell lysates prepared from newly constructed F15 strains. 1 mM IPTG added to the LB medium caused the *fis* overexpression in the all studied F15 originated strains (Fig 5). The *lapF* expression in the constructed strains was examined with silver-staining of SDS-polyacrylamide gels where proteins from cell lysates were separated by gel-electrophoresis (Fig 3B). LapF expression was downregulated in F15Km by the *fis* overexpression (Fig 3B) similarly to F15 [22]. Thus, the insertion of Km-resistance gene in front of the *lapF* gene did not affect the regulation of *lapF* expression by Fis. However, *lapF* was expressed in F15lapF3 and F15KmFm, in the strains missing correct Fis binding site Fis-F2, despite the *fis* overexpression (Fig 3B). These results confirmed that Fis repressed *lapF* expression via the Fis binding site Fis-F2. This is in good accordance with the previously published data showing that Fis binds to the Fis-F2 site upstream of the *lapF* gene and represses the transcription of *lapF* [32].

**Fig 6:**
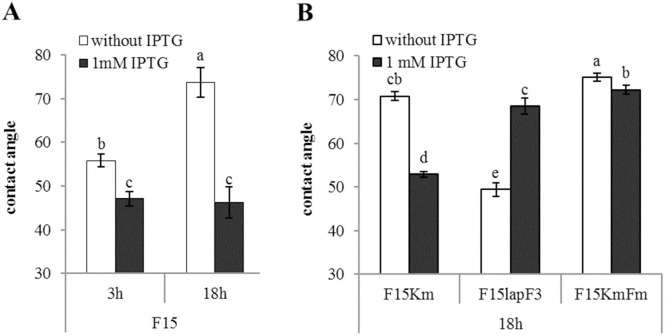
The cell surface hydrophobicity of *P*. *putida* F15-originated strains. (A) Hydrophobicity of exponential phase (3h) and stationary-phase (18h) *P*. *putida fis* overexpression strain F15 cultivated in the presence or absence of 1 mM IPTG in rich medium. (B) Hydrophobicity of stationary-phase cells (18h) of the *P*. *putida fis* overexpression strains expressing *lapF* at natural level (strain F15Km), under the control of the IPTG-inducible P_*tac*_ promoter (strain F15lapF3) and carrying mutations in the Fis binding site in the promoter region of the *lapF* gene (strain F15KmFm). Bacteria were grown in the presence or absence of 1 mM IPTG in rich medium. The level of cell surface hydrophobicity is given as water contact angles (θ_w_ in degrees) between water droplets and a filter covered with bacterial cells. Error bars denote 95% confidence intervals of the means. Letters a–e depict statistical homogeneity groups according to ANOVA *post hoc* Bonferroni test. Data from at least eight measurements is shown.

The hydrophobicity of *fis* overexpression strain F15 was measured when cells were grown in LB medium for 3 h (exponential phase) and 18 h (stationary phase) in the presence or absence of 1 mM IPTG (Fig 6A). We observed that the *fis* overexpression in the presence of IPTG decreased hydrophobicity of cells in exponential growth phase 1.2 times (*P*˂0.001) from θ_w_ value of 56° to 48° and in stationary growth phase cells 1.6 times, from θ_w_ 74° to 46° (*P*˂0.001; Fig 6A). Similarly to the original strain F15, the contact angle of the strain F15Km (θ_w_ 71°) decreased 1.3 times in stationary phase by the addition of IPTG to the medium (*P*˂0.001) comparing to F15Km grown without IPTG (θ_w_ 53°; Fig 6B). At the same time, the contact angle of the strain F15KmFm (θ_w_ 75°) decreased minimally by the addition of IPTG to the LB medium (*P* = 0.027) comparing to F15KmFm grown without IPTG (θ_w_ 72°; Fig 6B). On the contrary, measurement of the hydrophobicity of the cells of the strain F15lapF3 carrying the *lapF* gene under the control of the IPTG-inducible promoter revealed the increased hydrophobicity in the presence of IPTG. The contact angles increased 1.4 times to θ_w_ 68° when IPTG was added to the medium compared to the F15lapF3 grown without IPTG (θ_w_ 49°, *P*˂0.001; Fig 6B). This data indicates that the hydrophobicity of *P*. *putida* depends on the presence of LapF on the cell surface. The expression of *lapF* is in turn repressed by Fis via binding to the Fis-F2 site at the *lapF* promoter region. Furthermore, this assures that *fis* overexpression can be directly related to only one certain phenotype–change of the hydrophobicity of *P*. *putida* by explicitly regulating the transcription of *lapF* gene. Contrary to the previously published studies about the relationships of *P*. *putida* biofilm and hydrophobicity, the findings of the present study suggest that biofilm, especially the Fis-enhanced, and the LapF-mediated hydrophobicity are not directly connected with each other in *P*. *putida*.

### Flow cytometry analysis revealed protective role of LapF against methanol toxicity

Our results demonstrated that the expression of *lapF* increased the hydrophobicity of cell surface; therefore, we asked whether LapF could affect the viability of *P*. *putida* in the presence of hydrophilic and hydrophobic compounds like methanol and 1-octanol. Thus, the effects of methanol as an example for a hydrophilic solvent and 1-octanol as an example for a hydrophobic solvent to the membrane of *P*. *putida* wild-type strain PSm and the *lapF* knock-out strain PSmΔ*lapF* were investigated. The strain PSmlapF3 grown with IPTG was used as positive control to the wild-type strain PSm. Overnight grown cells treated with different concentrations of methanol and 1-octanol were stained using the LIVE/DEAD BacLight kit (Invitrogen) and flow cytometry analysis was used to determine the distribution of subpopulations.

When cells were treated with methanol at concentration of 25% and 50% (v/v) the survival of strains expressing LapF (PSm and PSmlapF3) was higher than the *lapF* knock-out mutant PSmΔ*lapF* (Table 4, S1 Fig). Contrary to the results obtained with methanol, in cells treated with 1-octanol at concentrations of 0.015% and 0.045% (v/v), the viability of cells lacking LapF increased (Table 4, S1 Fig). As this method allowed us to observe cytoplasmic membrane integrity, we can see that LapF, increasing cell surface hydrophobicity, can be an important defensive factor for bacteria against methanol as an example of hydrophilic compounds.

**Table 4:** Effect of LapF on cell membrane integrity in the presence of methanol and 1-octanol.

**Strain**	**IPTG**	**Methanol(%)**	**% of subpopulations**^***a***^		
			**Cells with intact membrane**	**Cells with permeable membrane**	**Dead cells**
PSm	0	0	93.6 (0.925)	1.82 (0.410)	4.56 (0.516)
	0	25	20.9 (3.370)	25.7 (1.964)	53.4 (3.535)
	0	50	0.08 (0.062)	50.6 (1.515)	49.3 (1.463)
PSmΔ*lapF*	0	0	97.2 (0.654)	0.61 (0.111)	2.18 (0.545)
	0	25	6.71 (0.422)	22.8 (1.949)	70.5 (2.244)
	0	50	0.27 (0.417)	7.97 (1.504)	91.8 (1.111)
PSmlapF3	1	0	97.2 (0.144)	0.39 (0.038)	2.40 (0.108)
	1	25	11.4 (0.251)	18.9 (0.594)	69.7 (0.413)
	1	50	0.25 (0.280)	33.7 (3.168)	66.0 (3.444)
**Strain**	**IPTG**	**1-octanol(%)**	**% of subpopulations**^***a***^		
			**Cells with intact membrane**	**Cells with permeable membrane**	**Dead cells**
PSm	0	0	95.6 (2.199)	1.22 (0.702)	3.22 (1.504)
	0	0.015	60.9 (0.885)	4.93 (0.155)	34.2 (0.732)
	0	0.045	66.6 (0.359)	3.20 (0.091)	30.2 (0.429)
PSmΔ*lapF*	0	0	97.6 (10.57)	0.47 (1.771)	1.95 (10.39)
	0	0.015	86.7 (2.578)	1.16 (0.322)	12.1 (2.583)
	0	0.045	81.5 (3.789)	1.44 (0.323)	17.1 (3.571)
PSmlapF3	1	0	97.6 (10.57)	0.47 (1.771)	1.95 (10.39)
	1	0.015	76.4 (3.572)	1.54 (0.401)	22.1 (3.755)
	1	0.045	70.4 (3.680)	1.83 (0.360)	27.8 (3.492)

^*a*^ In the brackets are shown 95% confidence intervals of the means of at least three measurements

## Conclusions

This study demonstrates that LapF is the key factor of cell surface hydrophobicity of *P*. *putida* in the stationary phase, and that Fis regulates the hydrophobicity of cell surface in *P*. *putida* through the repression of *lapF*. We observed that the LapF-mediated hydrophobicity protects *P*. *putida* cells against methanol as an example of hydrophilic solvents. On the other hand, bacteria expressing *lapF* were more susceptible to 1-octanol as an example of a hydrophobic solvent.

## Supporting Information

S1 Fig Visualization of subpopulations by flow cytometry analysis. *P*. *putida* strains PSm and PSmΔ*lapF* was grown for 18 hours in LB medium and thereafter treated with 25% (v/v) methanol and 0.15% (v/v) 1-octanol for 30 minutes. *P*. *putida* strain PSmlapF3 was grown for 18hours in LB medium amended with 1 mM IPTG and treated with chemicals similarly as other strains. Each dot represents an event, analysed by flow cytometer, that has been excitated at 488 nm and respective fluorescence emission has been measured at 530 (30) and 616 (23) nm. Area of subpopulations of dead cells, cells with permeable membrane and intact membrane are shown.
(TIF)
