# Supplementary figures and images for: LapF and Its Regulation by Fis Affect the Cell Surface Hydrophobicity of Pseudomonas putida
Source: PLoS One. 2016 Nov 3;11(11):e0166078. doi: 10.1371/journal.pone.0166078 (PMC5094663; doi:10.1371/journal.pone.0166078)

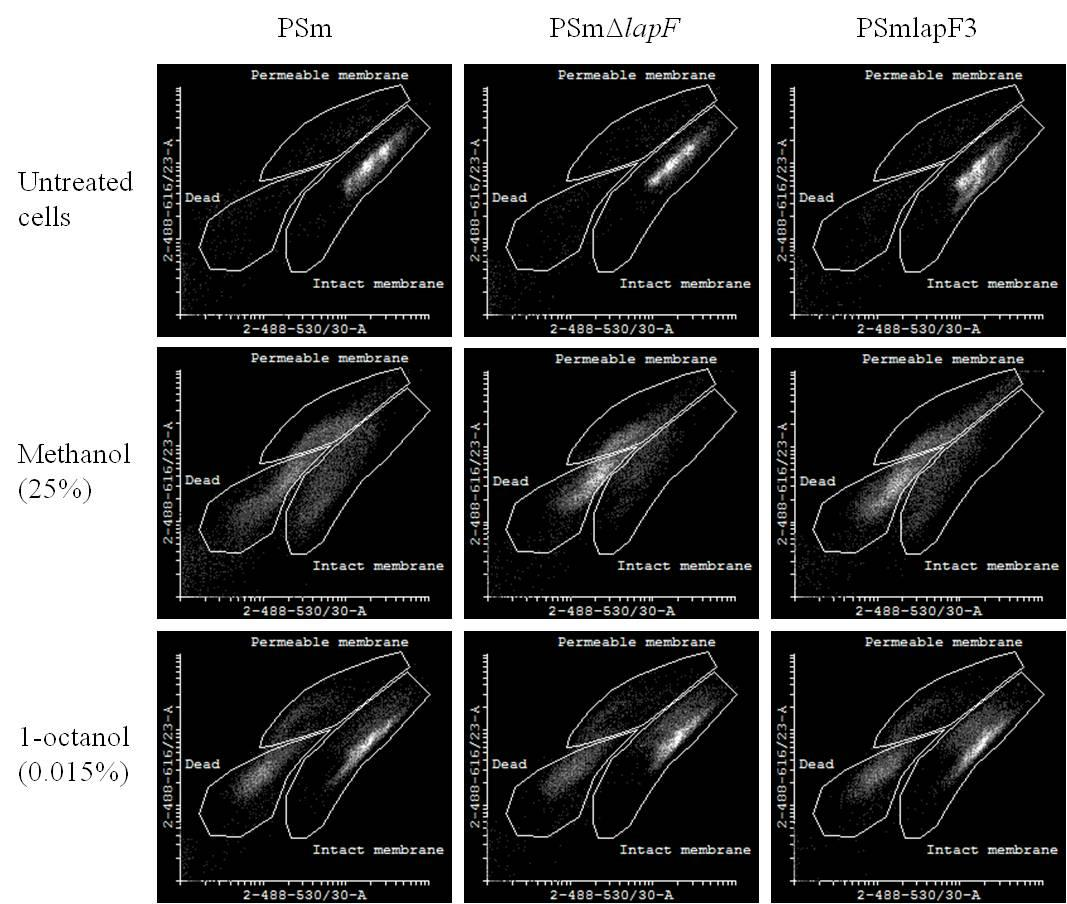

Supplement: S1 Fig — P. putida strains PSm and PSmΔlapF was grown for 18 hours in LB medium and thereafter treated with 25% (v/v) methanol and 0.15% (v/v) 1-octanol for 30 minutes. P. putida strain PSmlapF3 was grown for 18hours in LB medium amended with 1 mM IPTG and treated with chemicals similarly as other strains. Each dot represents an event, analysed by flow cytometer, that has been excitated at 488 nm and respective fluorescence emission has been measured at 530 (30) and 616 (23) nm. Area of subpopulations of dead cells, cells with permeable membrane and intact membrane are shown. (TIF) [file pone.0166078.s001.tif]
